# External evaluation of published population pharmacokinetic models of posaconazole

**DOI:** 10.3389/fphar.2022.1005348

**Published:** 2022-09-30

**Authors:** Shuqi Huang, Qin Ding, Nan Yang, Zexu Sun, Qian Cheng, Wei Liu, Yejun Li, Xin Chen, Cuifang Wu, Qi Pei

**Affiliations:** ^1^ Department of Pharmacy, The Third Xiangya Hospital, Central South University, Changsha, China; ^2^ Xiangya School of Pharmaceutical Sciences, Central South University, Changsha, China; ^3^ Department of Hematology, The Third Xiangya Hospital, Central South University, Changsha, China; ^4^ Center of Clinical Pharmacology, The Third Xiangya Hospital, Central South University, Changsha, China; ^5^ Department of Pharmacy, Affiliated Hospital of Guilin Medical University, Guilin, China

**Keywords:** posaconazole, population pharmacokinetics, external evaluation, predictive performance, therapeutic drug monitoring

## Abstract

Population pharmacokinetic (PopPK) models of posaconazole have been established to promote the precision dosing. However, the performance of these models extrapolated to other centers has not been evaluated. This study aimed to conduct an external evaluation of published posaconazole PopPK models to evaluate their predictive performance. Posaconazole PopPK models screened from the PubMed and MEDLINE databases were evaluated using an external dataset of 213 trough concentration samples collected from 97 patients. Their predictive performance was evaluated by prediction-based diagnosis (prediction error), simulation-based diagnosis (visual predictive check), and Bayesian forecasting. In addition, external cohorts with and without proton pump inhibitor were used to evaluate the models respectively. Ten models suitable for the external dataset were finally included into the study. In prediction-based diagnostics, none of the models met pre-determined criteria for predictive indexes. Only M4, M6, and M10 demonstrated favorable simulations in visual predictive check. The prediction performance of M5, M7, M8, and M9 evaluated using the cohort without proton pump inhibitor showed a significant improvement compared to that evaluated using the whole cohort. Consistent with our expectations, Bayesian forecasting significantly improved the predictive per-formance of the models with two or three prior observations. In general, the applicability of these published posaconazole PopPK models extrapolated to our center was unsatisfactory. Prospective studies combined with therapeutic drug monitoring are needed to establish a PopPK model for posaconazole in the Chinese population to promote individualized dosing.

## 1 Introduction

Posaconazole is commonly used for the prophylaxis or treatment of invasive fungal infections by inhibiting ergosterol biosynthesis in fungal cell membranes (US FDA, 2022; Wu et al., 2022), especially in patients such as acute myelogenous leukemia (AML), myelodysplastic syndrome (MDS), allogeneic hematopoietic stem cell transplantation, and other immunocompromised populations (Busca et al., 2021; Oh et al., 2021; Panagopoulou and Roilides, 2022). Posaconazole prophylaxis has the potential to reduce all-cause mortality and the incidence of adverse events in the aforementioned populations (Wang et al., 2020; Wong et al., 2020; Zeng et al., 2021).

Oral suspensions, delayed-release tablets, and injections are the three currently available formulations of posaconazole. Posaconazole oral suspension was approved by the U.S. Food and Drug Administration in 2006 for the prophylaxis of Aspergillus and Candida infections (US FDA, 2022). The effect of multiple factors on plasma exposure results in large inter-individual variability in posaconazole oral suspensions; however, posaconazole delayed-release tablets, approved in 2013, have improved the pharmacokinetics of posaconazole (Boglione-Kerrien et al., 2018; Gautier-Veyret et al., 2019; Sustained-Release, 2022). Nevertheless, it has been reported that high body weight, diarrhea, mucositis, hypoproteinemia, and concomitant use of proton pump inhibitors (PPIs), H_2_ receptor antagonists, and metoclopramide may lead to subtherapeutic concentrations of posaconazole oral suspensions and delayed-release tablets (Tang et al., 2017; Cojutti et al., 2018; Suh et al., 2018; Lai et al., 2020; Oh et al., 2020). Conversely, the intake of high-fat foods, nutritional supplements, and acidic beverages can facilitate the absorption of posaconazole oral suspension (Krishna et al., 2009). Intravenous formulation of posaconazole was approved in 2014, providing a new route of administration for patients with dysphagia and those in a coma (US FDA, 2022).

Posaconazole pharmacokinetics may be altered by inter- or intra-individual variability, formulations of posaconazole, and drug-drug interactions. Therapeutic drug monitoring (TDM) ensures optimal exposure to avoid breakthrough fungal infections or antifungal treatment failures resulting from low concentrations (Dolton et al., 2012; John et al., 2019). In addition, TDM is often combined with a population pharmacokinetic (PopPK) approach as a means of personalized medicine. PopPK, a mathematical modeling approach, is used to quantitatively assess the pharmacokinetic characteristics of absorption, distribution, metabolism, and excretion of an agent, as well as to explain inter-individual variability (Guidi et al., 2022). The validity and credibility of PopPK models can be evaluated with internal or external test datasets. Compared with internal evaluation, external evaluation is a rigorous and critical approach to examining the reproducibility and extrapolability of PopPK models for different patient populations and different scenarios (Siontis et al., 2015; Cheng et al., 2021; Ramspek et al., 2021).

In the last decade, PopPK models have been developed for posaconazole, incorporating multiple covariates to account for the variability in posaconazole exposure. However, external evaluation was often not performed for these models during their development. Thus, their predictive performance and applicability to other centers remain unexamined. Therefore, we conducted this study with the aim of evaluating published posaconazole PopPK models using an independent external dataset.

## 2 Materials and methods

### 2.1 Retrieval and screening of posaconazole PopPK models

Literature on posaconazole PopPK studies was searched in PubMed and MEDLINE databases until May 2022 using the following keywords: “posaconazole,” “population pharmacokinetic” and “nonlinear mixed effect model.” The reference lists of the selected publications were also inspected for a comprehensive search of published models.

Studies meeting the following criteria were included: 1) those conducted in humans; 2) those with posaconazole as the primary research drug; 3) those describing PopPK modeling of posaconazole. Literature with one of the following conditions was excluded: 1) review or external evaluation studies; 2) studies in which the available information was insufficient to reproduce the model; 3) studies including free and total plasma concentrations of posaconazole; 4) studies with non-compartment or non-parametric modeling methods.

### 2.2 External evaluation dataset

The retrospective external evaluation cohort consisted of patients who received posaconazole oral suspension at the Hematology Department of Third Xiangya Hospital of Central South University from 2019 to 2021 and had undergone TDM at least once. The dosage of posaconazole, ranging from 300 to 1,200 mg daily, was determined by a competent physician. Blood samples were collected intravenously before administration under the steady-state. Liquid chromatography-tandem mass spectrometry (LC-MS/MS) was used for the determination of posaconazole plasma concentrations with a linear range of 0.08–10 mg/L and an accuracy of 93.5%–106.04%. The following demographic and clinical information was collected from the electronic medical record system: age, sex, weight, gamma-glutamyl transferase (GGT), total protein, total bilirubin, albumin, and treatment information (disease diagnosis, purpose of medication, formulation, dosage, time of administration and sampling). In the absence of patient height data, body surface area (BSA) was estimated by Livingston et al.’s formula (Livingston and Lee, 2001): BSA (m^2^) = 0.1173 × weight (kg)^0.6466^. To explore the influence of the characteristics of the external cohort on the pharmacokinetics of posaconazole, we performed a statistical analysis of the correlation between sex, age, weight, diarrhea, PPI, and chemotherapy and plasma concentrations. Categorical variables (PPI, diarrhea, chemotherapy, sex) were presented as box plots, and continuous variables (age, body weight) were presented as scatter plots. This study was approved by the Ethics Committee of the Third Xiangya Hospital of Central South University. These data have not been used in the development of any models.

### 2.3 External evaluation of predictive performance of PopPK models

Parameter formulas and values were obtained from the published posaconazole PopPK models. Nonlinear mixed effect modeling (NONMEM version 7.5) software, assisted by Perl-Speaks-NONMEM (PsN, version 4.7.0; uupharmacometrics.github.io/PsN) and Pirana (version 2.9.2; www.pirana-software.com), was used for external evaluations, and R package (Version 4.1.2; http://www.r-project.org) was used for analysis and graphical processing of NONMEM output results.

#### 2.3.1 Prediction-based diagnosis

In prediction-based diagnostics, goodness-of-fit plots characterizing the fitness of observations versus population predictions and observations versus individual predictions were constructed to assess the predictive performance of the models. Prediction error (PE) was calculated with Eq. 1:

$$PE\%=\frac{PRED-OBS}{OBS}\times 100$$ (1)

where PRED is the concentration predicted using the final model and parameters in the literature, OBS is the concentration observed in the external evaluation data set.

PE is a classical prediction index of model accuracy and is presented as a boxplot in this study. Median prediction error (MPE) was used to examine the overall bias of models, with MPE >0 and MPE <0 indicating the tendency of the model to overestimate and underestimate the predicted value, respectively. The median absolute prediction error (MAPE) was used to characterize the precision of the models. Composite metrics F_20_ and F_30,_ the percentage of PE between ±20% and ±30%, simultaneously characterize the accuracy and precision of model predictions. Models with MPE ≤ ±20%, MAPE ≤30%, F_20_ ≥ 35%, and F_30_ ≥ 50% are considered acceptable (Cai et al., 2020; Li et al., 2021).

#### 2.3.2 Simulation-based diagnosis

The concordance between simulations and observations was demonstrated by a visual predictive check (VPC). Based on the observed data and the published model, 1,000 simulations were executed in NONMEM to generate the simulation dataset. The 95% confidence intervals were calculated for the 5th percentile, median, and 95th percentile of the simulated concentrations and compared to the percentile of the corresponding observed concentrations. The distribution characteristics of the observed and simulated data have been displayed as VPC plots using R software.

#### 2.3.3 Bayesian forecasting

Maximum *a posteriori* Bayesian estimation (MAPB) was performed to assess the impact of prior observations on the predictability of the model using 0, 1, 2, and 3 previous observations to predict patients with ≥1 observation. In the NONMEM control file, $POSTHOC item ' MAXEVALS ′ was set to 0. R software was used to process the output results and generate the graphics. Individual prediction for the last observation of all subjects was predicted by the latest 1, 2, and 3 prior observations, respectively. The individual prediction error (IPE) and absolute individual prediction error (AIPE) were calculated according to the individual predicted concentrations (IPRED) and observed concentrations. The formulae are shown as Eqs 2, 3 below:

$${IPE}_{i}\left(\%\right)=\frac{I{PRED}_{i}-{OBS}_{i}}{{OBS}_{i}}\times 100$$ (2)

$${AIPE}_{i}\left(\%\right)=\left|\frac{{IPRED}_{i}-{OBS}_{i}}{{OBS}_{i}}\right|\times 100$$ (3)

Median individual prediction error (MIPE) and median absolute individual prediction error (MAIPE) were used to evaluate the accuracy and precision of Bayesian forecasting, respectively. IF_20_ and IF_30_, which represented F_20_ and F_30_ of IPE, were also calculated (Zhao et al., 2016; Hanafin et al., 2021). MIPE% ≤ ± 20%, MAIPE% ≤ 30%, IF_20_ ≥ 35% and IF_30_ ≥ 50% were used to assess the predictive performance of the model when adding the prior concentrations.

## 3 Results

### 3.1 Review of published PopPK models of posaconazole

After retrieval and screening, 10 PopPK models of posaconazole (Abutarif et al., 2010; Störzinger et al., 2012; Vehreschild et al., 2012; Dolton et al., 2014; Petitcollin et al., 2017; Boonsathorn et al., 2019; Wasmann et al., 2020; Bentley et al., 2021; Elkayal et al., 2021; Peña-Lorenzo et al., 2022) published from 2010 to 2021 were finally included in our study. There were six single-center studies (Störzinger et al., 2012; Vehreschild et al., 2012; Petitcollin et al., 2017; Boonsathorn et al., 2019; Bentley et al., 2021; Peña-Lorenzo et al., 2022) and four multi-center studies (Abutarif et al., 2010; Dolton et al., 2014; Wasmann et al., 2020; Elkayal et al., 2021). In these studies, six were adult healthy volunteers or patients (Störzinger et al., 2012; Vehreschild et al., 2012; Dolton et al., 2014; Petitcollin et al., 2017; Wasmann et al., 2020; Peña-Lorenzo et al., 2022), three were pediatric patients (Boonsathorn et al., 2019; Bentley et al., 2021; Elkayal et al., 2021), and one included both adults and pediatric patients (Abutarif et al., 2010). The majority of the population included in the studies were patients with neutropenic hematologic diseases such as AML, MDS, and stem cell transplantation. Three studies were performed with a large sample size of over 100 subjects (Abutarif et al., 2010; Dolton et al., 2014; Boonsathorn et al., 2019). Except for one study (Wasmann et al., 2020) involving intravenous administration of posaconazole, patients in the other studies were administered posaconazole via the gastrointestinal tract in the form of oral suspensions or delayed-release tablets. Almost all plasma samples of these studies were determined with liquid chromatography or LC-MS/MS. Half of the studies (Abutarif et al., 2010; Vehreschild et al., 2012; Dolton et al., 2014; Boonsathorn et al., 2019; Elkayal et al., 2021) incorporated diarrhea and PPI into their final models, and three studies (Vehreschild et al., 2012; Boonsathorn et al., 2019; Wasmann et al., 2020) incorporated body weight. Few studies retained sex, race, total protein, bilirubin, GGT, mucositis, and concomitant medications such as chemotherapy, phenytoin, rifampin, fosamprenavir, nutritional supplements, and metoclopramide in the final model. The characteristics of the published models were summarized in Table 1.

**TABLE 1:** Summary of published population pharmacokinetic studies of posaconazole.

Study	Country (year)	Study design	Subject characteristic	N (male/female)	Age (year)^a^	Body weight (kg)^a^	Number of observations	Route	Structural model	PK formulas and parameters	IIV% (IOV%)	RV
M1 (Abutarif et al., 2010)	US (2010)	Multi-center, P	Adult & pediatrics, neutropenic patients receiving chemotherapy for AML/MDS	215 (117/98)	52	70	702	Oral suspension	1-CMT	CL/F = 65.1	V/F: 15.6	1.03%
										V/F = 3,290 × 1.5^diarrhea*^ ×1.43^PPI*^× 1.84^bilirbin*^ ×1.17^GGT*^ ×0.79^race*^		
										K_a_ = 0.0396	K_e_: 2.21	
										K_e_ = 0.0198		
M2 (Vehreschild et al., 2012)	Germany (2012)	Single-center, P	Adult, patients with AML/MDS	84 (42/42)	55 (19–73)	77.7 (48.0–119.2)	643	Oral suspension	1-CMT	CL/F = 42.5×θ_PPI_^PPI*^× θ_Di_^diarrhea*^	CL/F: 25.3	23.2%
										V/F = [2,770+(WT-78) ×θ_WT_] × θ_CHEM_^CHEM*^		
										K_a_ = 0.4 (fixed)		
M3 (Störzinger et al., 2012)	Germany (2012)	Single-center, P	Adult, patients in a SICU	15 (6/9)	58 (41–79)	NA	270	Nasogastric tube	1-CMT	CL/F = 195	CL/F: 51.8 (48.4)	11.6%
										V/F = 5,280		
										K_a_ = 0.77	V/F: 52.0 (21.1)	2.8%
M4 (Dolton et al., 2014)	Australia, Netherlands (2014)	Multi-center, R	Adult, healthy volunteers (study1) & patients (study2)^b^	102 (58/44)	Study1	Study1	905	Oral suspension	1-CMT	CL/F = 30.2 × 7.21^PHE*^× 7.21^RIF*^ ×1.342^FOS*^	CL/F: 46.4	6.76% (study1)
					38 (18–54)	74 (44–104)				V/F = 1,100	V/F: 30.2	
					Study2	Study2				K_a_ = 1.26	K_a_: 53.4	53.8% (study2)
										T_lag_ = 1.79		
					50 (18–79)	71 (38–122)				F = 0.549^PPI*^ × 0.655^MET*^ × 2.29^NUT*^ × 0.423^MUC*^ × 0.549^diarrhea*^	F: (23.6)	
M5 (Petitcollin et al., 2017)	France (2017)	Single-center, P	Adult, hematological malignancies	49 (29/20)	53 (19–73)	72 (50–125)	205	Tablet	1-CMT	CL/F = 7.3 L/h	CL/F: 24.2 (31.9) V/F: 28.2	14.8%
										V/F = 420 L		
										K_a_ = 0.588h^−1^ (fixed)		
M6 (Boonsathorn et al., 2019)	UK (2019)	Single-center, R	Infants & Children, immunocom-promised	117 (43/74)	5.7 (0.5–18.5)	17.8 (6.05–74.8)	338	Oral suspension & Tablet	1-CMT	CL/F = 14.95×(WT/70)^0.75^	CL/F: 63.0	47.29% 0.02 mg/L
										V/F = 201.7×(WT/70)		
										β_dose_ = 99 mg/m^2^ (fixed)		
										K_a_ = 0.588× (WT/70)^−0.25^ (fixed) (suspension)		
										F = 1 (suspension)		
										K_a_ = 0.197× (WT/70)^−0.25^ (fixed) (tablet)		
										F = [1 - D/(D + β_dose_)] × 0.67^diarrhea*^ × 0.58^PPI*^(tablet)		
M7 (Wasmann et al., 2020)	Netherlands (2020)	Multi-center, P	Adult, obese & non-obese healthy volunteers	24 (12/12)	Normal (300 mg IV): 22 (20–37); Obese (300 mg IV): 51 (31–63); Obese (400 mg IV): 37.5 (25–50)	Normal (300 mg IV):72.3 (61.4–85.4)	226	IV	2-CMT	CL = 5.83×(TBW/70)^0.54^	V_1_: 29.5	16.4%
						Obese (300 mg IV): 129 (109–190)				Q = 60.3		
										V_1_ = 150×(TBW/70)^0.77^		
						Obese (400 mg IV): 144 (107–175)				V_2_ = 96.2×(TBW/70)^1.16^		
M8 (Peña-Lorenzo et al., 2022)	Spain (2021)	Single-center, P	Adult, SCT recipients	36 (17/19)	53 (27–73)	68.3 (40.0–103.5)	55	Tablet	1-CMT	CL/F = 8.02 × 0.613^SEX*^ ×(PROT/6.4)^−1.48^	CL: 28.9	21.6%
										V/F = 548		
										K_a_ = 0.795 h^−1^ (fixed)	V: 52.4	
										D1 = 2.62 h (fixed)		
M9 (Bentley et al., 2021)	UK (2021)	Single-center, R	Pediatrics, cystic fibrosis	37 (13/24)	14 (7–17)	45.55 (25–82.8)	100	Tablet	1-CMT	CL/F = 8.43	CL/F: 38.0	36.0% 0.15 mg/L
						Age 6–11 years						
						31.5 (25–58)				V/F = 186		
						Age 12–17 years						
						50 (34.7–82.8)				K_a_ = 0.16		
M10 (Elkayal et al., 2021)	Romania (2021)	Multi-center, P	Pediatrics, hematologic malignancies	14 (5/9)	6.7 ± 2.8	19.9 ± 6.1	112	Oral suspension	1-CMT	CL/F = 15.4×(WT/70)^0.75^	CL/F: 87.8	11.0%
										V/F = 1,150×(WT/70)		
										K_a_ = 0.325× (WT/70)^0.25^		
										T_lag_ = 2.71		
										β_dose_ = 99.1mg/m^2^ (fixed)		
										F = [1 - D/(D + β_dose_)] ×0.67^diarrhea*^×0.58^PPI*^		

a Values are expressed as median (range), mean (range) or mean ± standard deviation.

b Patients with the underlying condition: AML; acute lymphoblastic leukemia; Non-Hodgkin’s lymphoma; MDS; multiple myeloma; Diabetes mellitus type 2; Chronic lymphocytic leukemia; Myelofibrosis; Hodgkin’s lymphoma; acute biphenotypic leukemia; gray-zone lymphoma; T-polylymphocytic leukemia; chronic myeloid leukemia; aplastic anemia; HIV positivity; rheumatoid arthritis; Crohn’s disease; and none (histoplasma).

* diarrhea/PPI/CHEM/PHE/RIF/FOS/MET/NUT/MUC = 0 in the absence of this covariate, diarrhea/PPI = 1 in the presence of this covariate; bilirubin = 0 if the bilirubin levels<2×ULN, bilirubin = 1 if the bilirubin levels≥2×ULN; GGT = 0 if the GGT levels<2×ULN, GGT = 1 if the GGT levels≥2× ULN; race = 0 if the patient is nonwhite, race = 1 if the patient is white; SEX = 0 for men and SEX = 1 for women.

P, prospective; R, retrospective; AML, acute myelogenous leukemia; MDS, myelodysplastic syndrome; SICU, surgical intensive care unit; SCT, allogeneic stem cell transplant; IV, intravenous administration; CMT, compartment; PK, pharmacokinetic; CL/F, apparent oral clearance from whole blood; V/F, apparent oral volume of distribution in whole blood; K_a_, absorption rate constant; K_e_, elimination rate constant; Tlag, absorption lag time; F, bioavailability; βdose, estimated dose in mg/m^2^ for suspension bioavailability to drop to half that of the tablet; D, dose in mg/m^2^; CL, clearance; Q, intercompartmental clearance; V_1_, central volume of distribution; V_2_, peripheral volume of distribution; D1, duration of zero-order absorption into depot compartment; PPI, proton pump inhibitor; GGT, gamma-glutamyl transferase; WT, weight; CHEM, co-administration of chemotherapy; PHE, phenytoin; RIF, rifampin; FOS, fosamprenavir; MET, metoclopramide; NUT, nutritional supplement; MUC, mucositis; TBW, total body weight; SEX, sex; PROT, total proteins; IIV, inter-individual variability; IOV, inter-occasion variability; RV, residual variability.

### 3.2 External evaluation data set

After removing the 4.48% (10/223) of samples with concentration below limit of quantification according to the method of Irby et al. (2021), 213 trough concentrations at the steady-state of posaconazole from 97 (58 males and 39 females) patients were finally retained. The median (range) of posaconazole concentration was 0.77 (0.08–2.76) mg/L. Concomitant medications of PPI and chemotherapy agents were administrated in 74% and 38% of patients during the treatment with posaconazole, respectively. Only a few patients received a combination of nutritional supplements, phenytoin, and rifampicin. In addition, 12% of patients developed diarrhea during treatment. Detailed demographic information and clinical data were summarized in Table 2.

**TABLE 2:** Summary of the demographic information and clinical data of the external data set.

Characteristics	Data
Demographic data	
Number of patients	97
Age (years)	46 (12–83)^a^
Sex (male/female, n)	58/39
Body weight (kg)	59 (31–90)^a^
Body surface area (m^2^)^b^	1.64 (1.08–2.15)^a^
Pharmacokinetic data	
Number of posaconazole concentrations	213
Posaconazole concentration (mg/L)	0.77 (0.08–2.76)^a^
Time of sampling after dosing (h)	13.38 (7–47)^a^
Samples per patient	2 (1–10)^a^
Dose (mg/dose)	200 (100–400)^a^
Biological and clinical data	
GGT	43 (11–97)^a^
Total protein (g/L)	6.27 (4.64–10.5)^a^
Bilirubin	11 (3.6–78.3)^a^
Diarrhea^c^	12
Coadministered agents^c^	
Proton pump inhibitor (n)	72
Chemotherapy^c^	37
Nutritional supplement (n)	8
Metoclopramide (n)	0
Phenytoin (n)	2
Rifampin (n)	1
Fosamprenavir (n)	0

a Presented as median (range).

b Estimated BSA = 0.1173×WT (kg)^0.6466^, where BSA is body surface area (m^2^), WT is body weight.

c Presented as number of patients.

AML, acute myelogenous leukemia; MDS, myelodysplastic syndrome.

Statistical analysis showed significant differences in the plasma concentrations of posaconazole in subjects with and without PPI (*p* = 0.0013), as well as diarrhea (*p* = 0.0044). There was a weak positive correlation between body weight and posaconazole concentrations (R = 0.15, *p* = 0.029). The box plot and scatter plot results were shown in the Supplementary Figure S1. Therefore, we divided the evaluation dataset into two parts by subjects with or without PPI during posaconazole treatment. Due to changes in PPI use during treatment, some patients were double counted (*n* = 11) and some samples were removed (N = 16). Finally, 139 plasma samples from 71 patients were assigned to the external cohort with PPI (cohort 1), and 58 plasma concentrations from 37 patients were assigned to the external cohort without PPI (cohort 2). The whole cohort was used for the evaluation of all models included, and cohort 1 and cohort 2 were used for the evaluation of models that included PPI and models that did not include PPI, respectively. The models were not evaluated in groups by diarrhea because the number of patients with diarrhea in the external data was only 12.4%.

### 3.3 Results of external evaluation

#### 3.3.1 Prediction-based diagnosis

**FIGURE 1:**
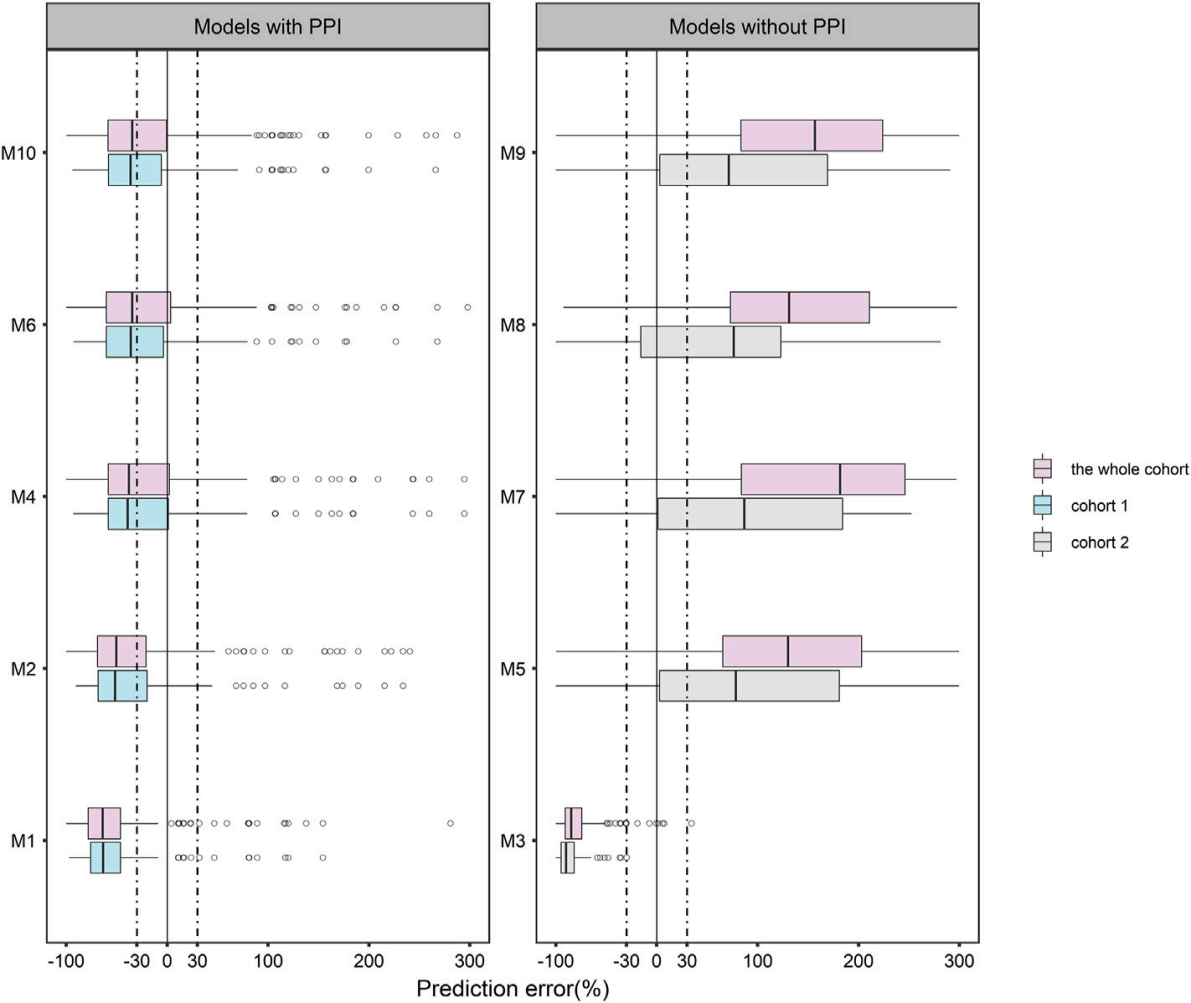
Boxplots of prediction error (PE) for 10 published population pharmacokinetic models of posaconazole.

The goodness-of-fit test results for the prediction error diagnostics using the entire cohort, cohort 1 and cohort 2, were presented in Supplementary Figures S2–S9. The box plots of the prediction errors were shown in Figure 1. In terms of accuracy and precision, M4, M6, and M10 were superior to other models. Regardless of which cohort was used for evaluation, no model had a median prediction error within an acceptable range of ±30%. In addition, MPE, MAPE, F_20_, and F_30_ for the 10 models were not within the acceptable range according to the predetermined criteria (Supplementary Table S1). We observed that M5, M7, M8 and M9 had a higher median prediction error and a wider distribution than the other models. Compared to being evaluated with the whole cohort, prediction errors were significantly lower for M5, M7, M8, and M9 when evaluated with cohort 2. No noticeable changes in diagnostic outcomes were observed for the other models.

#### 3.3.2 Simulation-based diagnosis

**FIGURE 2:**
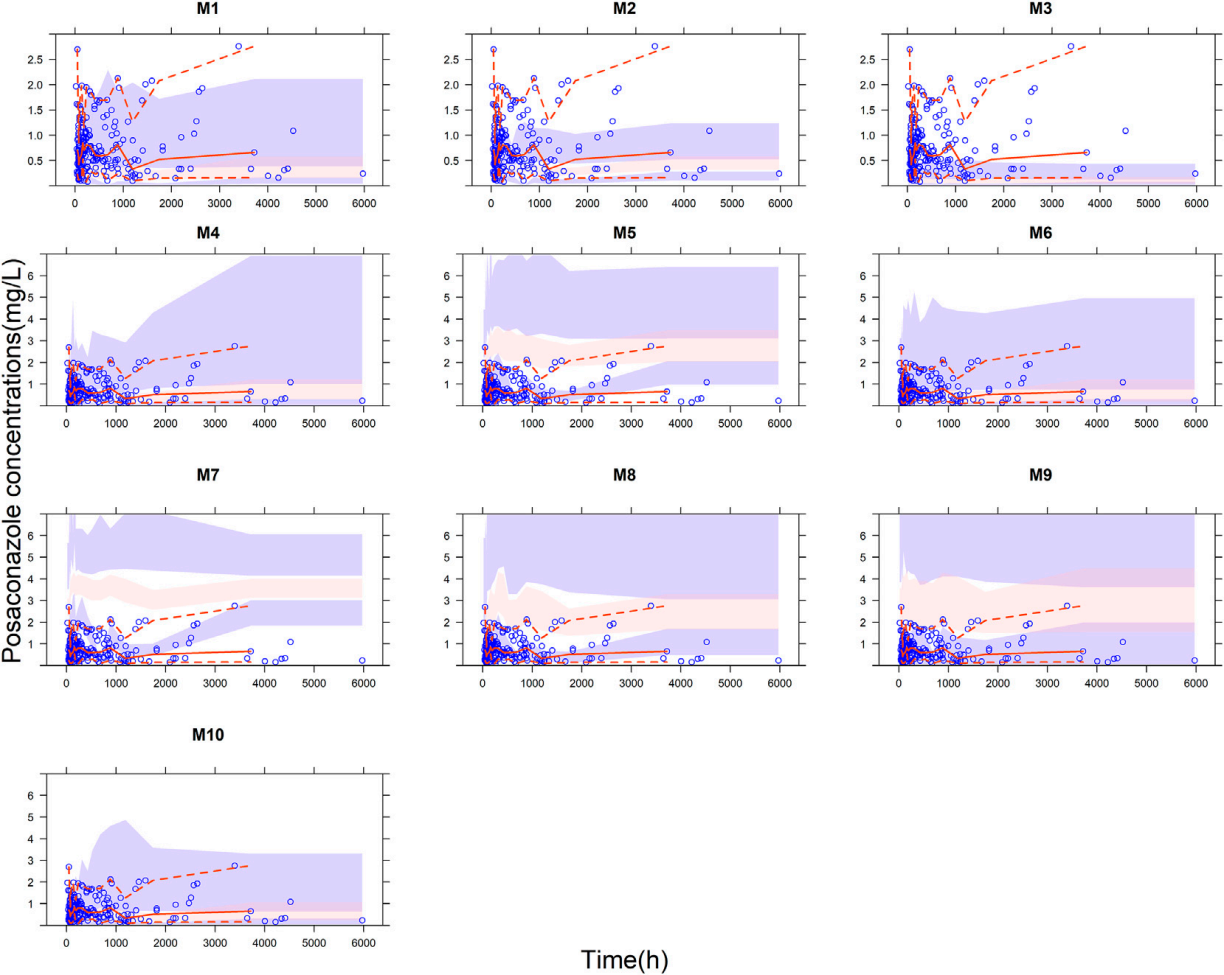
Visual predictive check (VPC) plots for the published model of posaconazole evaluated with the whole cohort. Blue points represent the observations, and red lines represent the 5th, 50th and 95th percentiles of the observed data. The color-shaded areas represent the 95% confidence intervals around the simulated 5th, 50th, and 95th percentiles.

The VPC results of each model were shown in Figure 2. The 5th, 50th, and 95th percentiles of most of the observed data of M4 (Dolton et al., 2014), M6 (Boonsathorn et al., 2019), and M10 (Elkayal et al., 2021) fall within the 95% prediction interval of the corresponding percentile of the simulated data. In addition, M2 (Vehreschild et al., 2012) and M9 (Bentley et al., 2021) suggest that the observed data had similar distribution characteristics to the simulated data near the 5th percentile. However, none of the other models fit well. The VPC results evaluated by PPI grouping were shown in the Supplementary Figure S10.

#### 3.3.3 Bayesian forecasting

**FIGURE 3:**
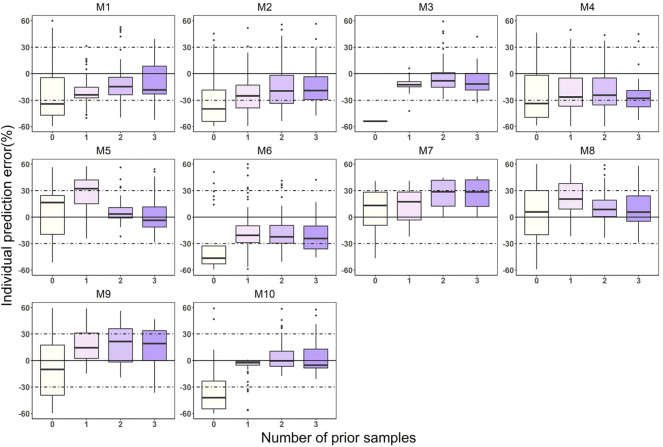
Box plots of individual prediction error (IPE) with Bayesian forecasting for 10 published PopPK models of posaconazole in different occasions. 0 represents predictions without prior information and 1-3 represents with prior one to three observations, respectively.

The relationship between IPE and the number of prior samples was shown in Figure 3. The results indicated that prior exposure can significantly reduce the prediction error of the models. Overall, the individual predictive performance of the models was optimal with two or three prior collections. With the addition of three prior concentrations, M1 (Abutarif et al., 2010), M2 (Vehreschild et al., 2012), M3 (Störzinger et al., 2012), M5 (Petitcollin et al., 2017), M8 (Peña-Lorenzo et al., 2022), and M10 (Elkayal et al., 2021) showed good accuracy and precision, meeting MIPE < ±20%, MAIPE <30%, IF_20_ > 35%, and IF_30_ > 50%. Especially in M3 (Störzinger et al., 2012) and M5 (Petitcollin et al., 2017), IF_30_ exceeded 70%. M3 (Störzinger et al., 2012) and M10 (Elkayal et al., 2021) achieved high predictive performance even with only one prior sample.

## 4 Discussion

PopPK models for posaconazole have been developed in some centers, although their applicability to other scenarios is unclear. This study provided the first external evaluation of ten published posaconazole PopPK models (Abutarif et al., 2010; Störzinger et al., 2012; Vehreschild et al., 2012; Dolton et al., 2014; Petitcollin et al., 2017; Boonsathorn et al., 2019; Wasmann et al., 2020; Bentley et al., 2021; Elkayal et al., 2021; Peña-Lorenzo et al., 2022) with an independent dataset to examine the clinical applicability of the models to our center and the heterogeneity among these models.

M4 (Dolton et al., 2014) presented a better predictive performance in the studies of adults than other models. Similar to M7 (Wasmann et al., 2020), M4 (Dolton et al., 2014) included both healthy volunteers and patients with various pathological states into the modeling population. Unlike other models, M4 (Dolton et al., 2014) retained most of the covariates examined in the final model under strict screening criteria, including phenytoin and rifampicin—inducers of the UDP - glucuronosyltransferases that mainly mediates the metabolism of posaconazole to posaconazole-glucuronide (Rae et al., 2001; Anderson, 2004; Ghosal et al., 2004).

For models developed with pediatric patients, both in prediction-based and simulation-based diagnostics, M6 (Boonsathorn et al., 2019) and M10 (Elkayal et al., 2021) were superior to M9 (Bentley et al., 2021), despite the PE being still slightly above 30%. Although only 16% (34/213) of the pediatric concentrations were included in the external evaluation dataset, we attempted to evaluate the models developed in the pediatric population to obtain a comparison between the adult and pediatric models. Studies have reported that rapid pharmacokinetic maturation tends to occur in the early stages from neonatal to infant (Rhodin et al., 2009; Germovsek et al., 2017; Salem et al., 2021). M6 (Boonsathorn et al., 2019) covers the population aged 0.5–18 years; however, the evaluation dataset lacks concentration data for children under 12 years of age, which may have an impact on the reliability of the evaluation results. M10 (Elkayal et al., 2021), a model developed with only 14 subjects, had large inter-individual variability (87.8%) in the estimated clearance, which may account for the poor predictive performance of the model. In addition, the dosing of M6 (Boonsathorn et al., 2019) and M10 (Elkayal et al., 2021) were based on BSA. However, the BSA for the evaluation data was estimated according to the formula provided in the literature. We have not been able to clarify the differences in the evaluation results caused by the different ways of obtaining BSA. M9 (Bentley et al., 2021) was established in patients with pulmonary fibrosis, while the evaluation population consisted mainly of patients with hematological diseases. Nevertheless, due to the limited number of published posaconazole PopPK models, we retained the models established in diverse populations. The difference in the pathological state between the modeling and the evaluation data may affect the evaluation of this model.

We observed that several models using tablets or intravenous preparations (M5, M7, M8, M9) (Petitcollin et al., 2017; Wasmann et al., 2020; Bentley et al., 2021; Peña-Lorenzo et al., 2022) showed a trend of overestimation and wide distribution of evaluation results compared to the models using oral suspensions. This may be related to the fact that the bioavailability of posaconazole varies considerably between formulations. The delayed-release tablets of posaconazole are less likely to recrystallize in the intestine due to the formulation improvements and are less affected by food, drugs that alter gastric pH and gastric motility, and therefore have a higher exposure compared to oral suspensions (Wiederhold, 2016; Van Daele et al., 2020). Since the population in the external dataset used oral suspensions, the accuracy and precision of the models for tablet and intravenous administration were not quite satisfactory.

In the external cohort, 74% (72/97) of patients were treated with concomitant PPI. Half of the models we evaluated included PPI as a covariate that could reduce posaconazole exposure. In the external cohort, plasma concentrations were significantly lower in patients with PPI than in those without PPI (*p* = 0.0013). The effect of concomitant PPI in the external cohort on the evaluation results of the models deserves to be considered. Therefore, we grouped the evaluation datasets according to the presence or absence of PPI. Compared to using the entire cohort, the results of the models evaluated with cohort 1 did not show significant differences, while the predictive performance of the models evaluated with cohort 2 showed a greater improvement. In particular, model improvements were more evident in the models using tablets and intravenous formulations (M5, M7, M8, M9), while little change in predictive performance could be observed in the models using oral suspensions. The predictions of the models that did not include PPI showed a higher trend when evaluated using the whole cohort. Furthermore, the number of patients with PPI was almost twice that of patients without PPI, which increased the impact of the evaluation cohort on the predictive performance of the models. This effect is mitigated when using cohort 1, so the prediction performance of the model is improved.

Except for the five models (Abutarif et al., 2010; Vehreschild et al., 2012; Dolton et al., 2014; Boonsathorn et al., 2019; Elkayal et al., 2021) that retained diarrhea and PPI, the other covariates differed considerably among models. As shown in the goodness-of-fit plots (Supplementary Figures S2–S5), the individual predicted values of some models were more consistent with the observed data than the population predicted values, which suggested that some potential covariates need to be further explored based on the structural model.

Based on the results of Bayesian forecasting, we can conclude that prior concentration can significantly improve the predictive performance of the model, which means that although these models are limited in the application of initial dose recommendation, they can play an important role in subsequent dose adjustment as long as two or three prior concentrations have been measured.

Nevertheless, the limitations of this study should be considered. First, the modeling population was mainly Caucasian, while the evaluation data were from Chinese patients. As reported by M1 (Abutarif et al., 2010), race may have an effect on posaconazole exposure, thus interfering with the veracity of the evaluation. Second, we must recognize that the variations between evaluation data and modeling data, including basic information on disease status, formulation, route of administration, concomitant medication and prandial state, to some extent, explained the results of unsatisfactory model evaluation. In addition, due to the limited data for the pediatric population in the evaluation dataset, the evaluation of models built with the pediatric patients may be less reliable.

## 5 Conclusion

External evaluation revealed that the currently published PopPK model has poor predictive performance for posaconazole concentrations and insufficient extrapolation to our center. Hence, it is necessary to establish a PopPK model of posaconazole for the Chinese population and to combine it with TDM for the recommendation and optimization of dosing regimens.

## Data Availability

The raw data supporting the conclusion of this article will be made available by the authors, without undue reservation.

## Abbreviations

PopPK, population pharmacokinetic; AML, acute myelogenous leukemia; MDS, myelodysplastic syndrome; PPI, proton pump inhibitors; TDM, therapeutic drug monitoring; LC-MS/MS, liquid chromatography-tandem mass spectrometry; GGT, gamma-glutamyl transferase; BSA, body surface area; NONMEM, nonlinear mixed effect modeling; PE, prediction error; PRED, predicted concentration; OBS, observation; MPE, median prediction error; MAPE, median absolute prediction error; F_20_, the percentage of prediction error falling within 20%; F_30_, the percentage of prediction error falling within 30%; VPC, visual predictive check; IPE, individual prediction error; AIPE, absolute individual prediction error; IPRED, individual predicted concentration; MIPE, median individual prediction error; MAIPE, median absolute individual prediction error; IF_20_, the percentage of individual prediction error falling within 20%; IF_30_, the percentage of individual prediction error falling within 30%.
