# Supplementary material for: External evaluation of published population pharmacokinetic models of posaconazole
Source: Front Pharmacol. 2022 Sep 30;13:1005348. doi: 10.3389/fphar.2022.1005348 (PMC9561726; doi:10.3389/fphar.2022.1005348)
Supplement: Supplementary file 1 [file DataSheet1.zip › Supplementary figures and tables.docx]

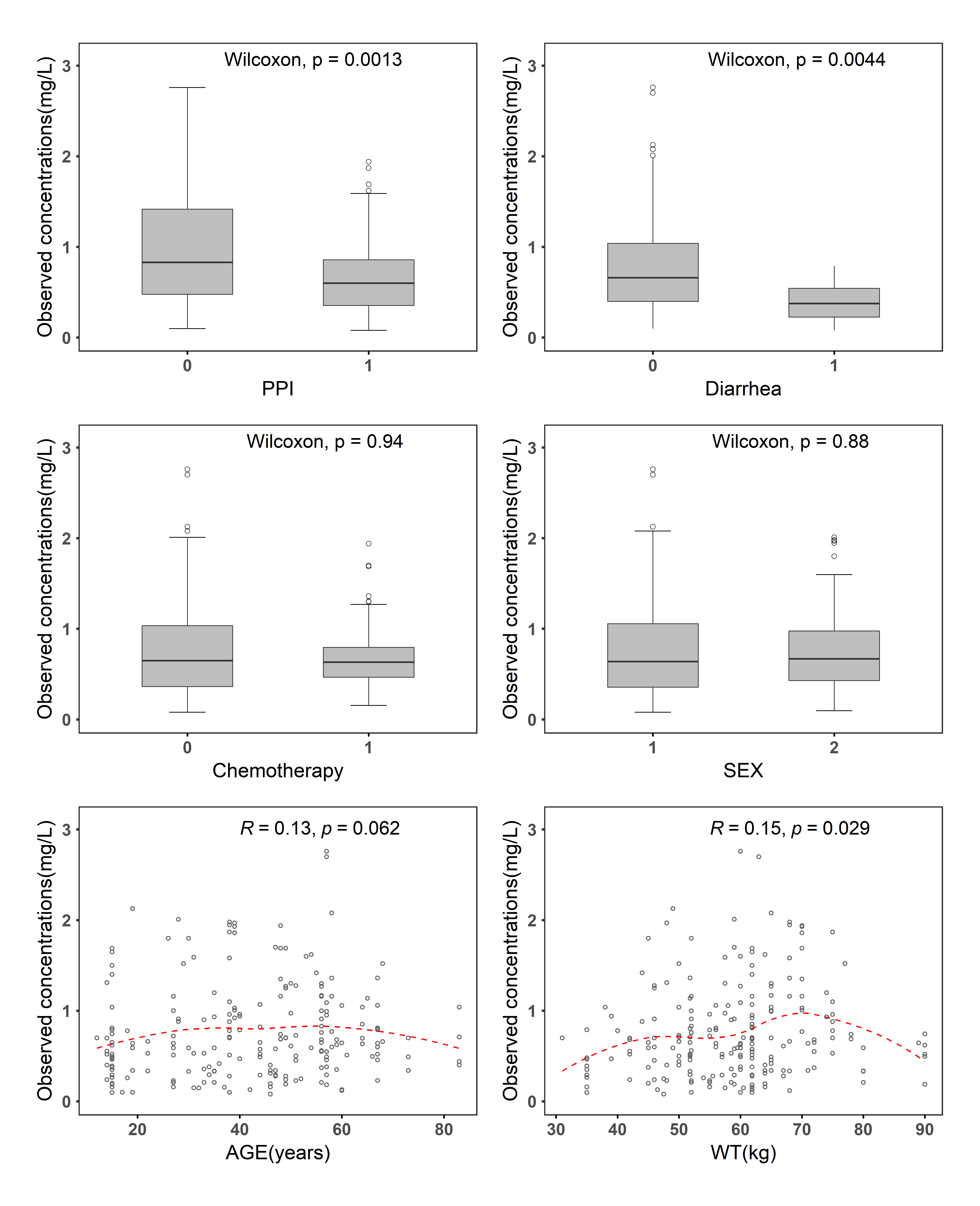


**Supplementary Figure 1.** Characteristics of the external cohort versus observed plasma concentrations of posaconazole.


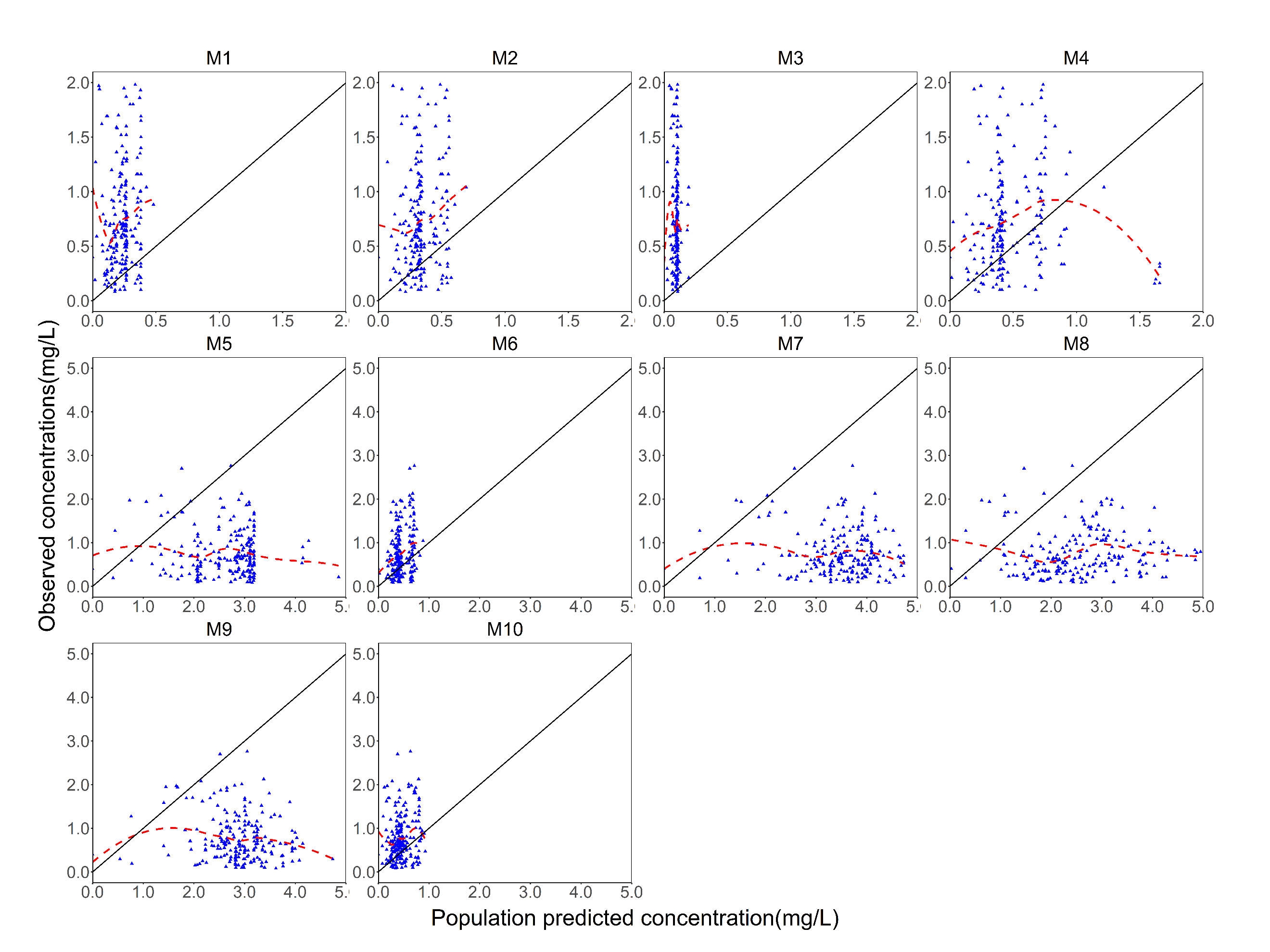


**Supplementary Figure 2.** Observed concentrations versus population predicted concentrations of the 10 models evaluated with the whole cohort.





**Supplementary Figure 3.** Observed concentrations versus population predicted concentrations of 10 models evaluated with cohort 1 (cohort with PPI treatment in the evaluation data) and cohort 2 (cohort without PPI treatment in the evaluation data).


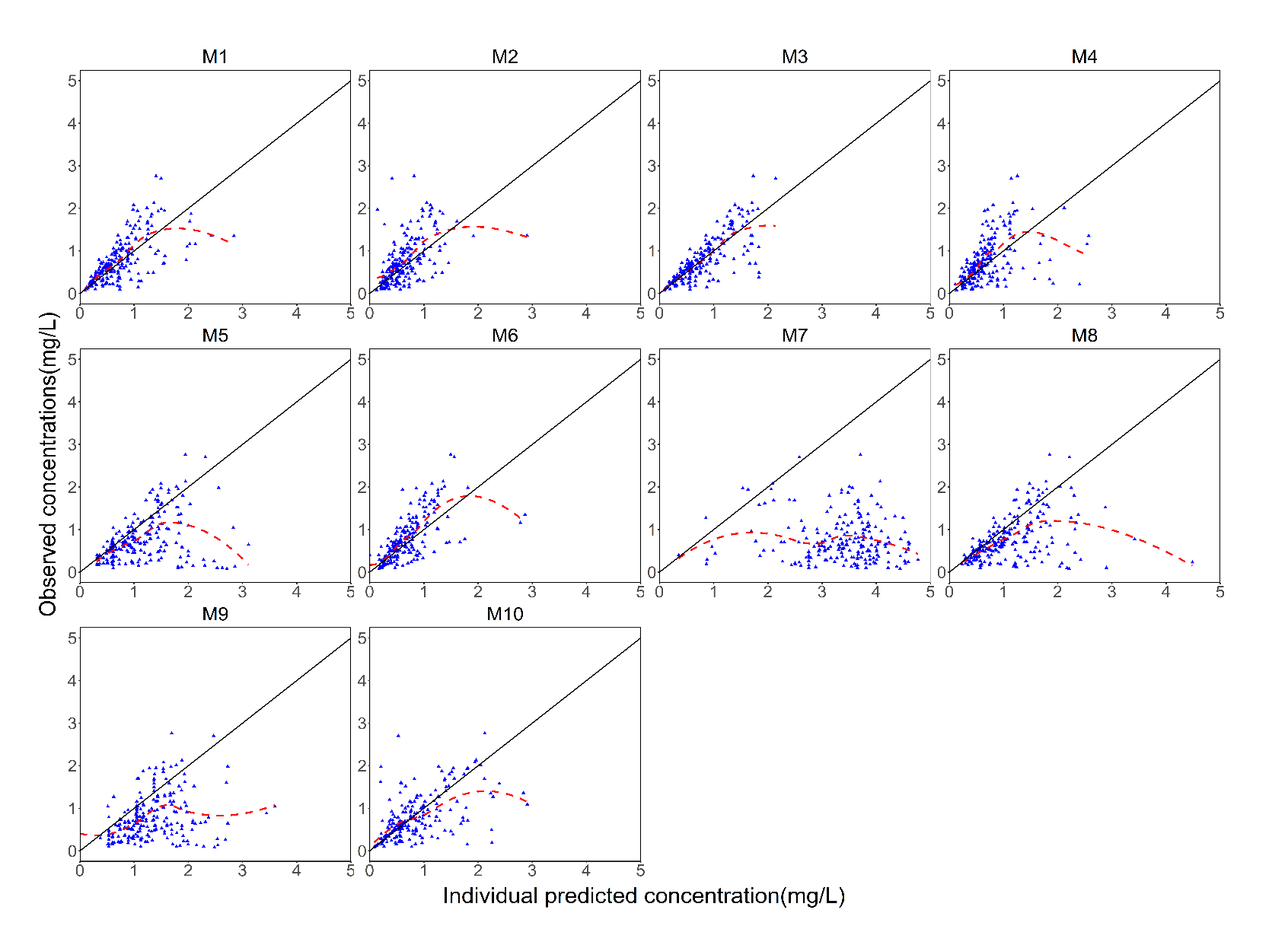


**Supplementary Figure 4.** Observed concentrations versus the individual predicted concentrations of 10 models evaluated with the whole cohort.





**Supplementary Figure 5.** Observed concentrations versus the individual predicted concentrations of 10 models evaluated with cohort 1 (cohort with PPI treatment in the evaluation data) and cohort 2 (cohort without PPI treatment in the evaluation data).


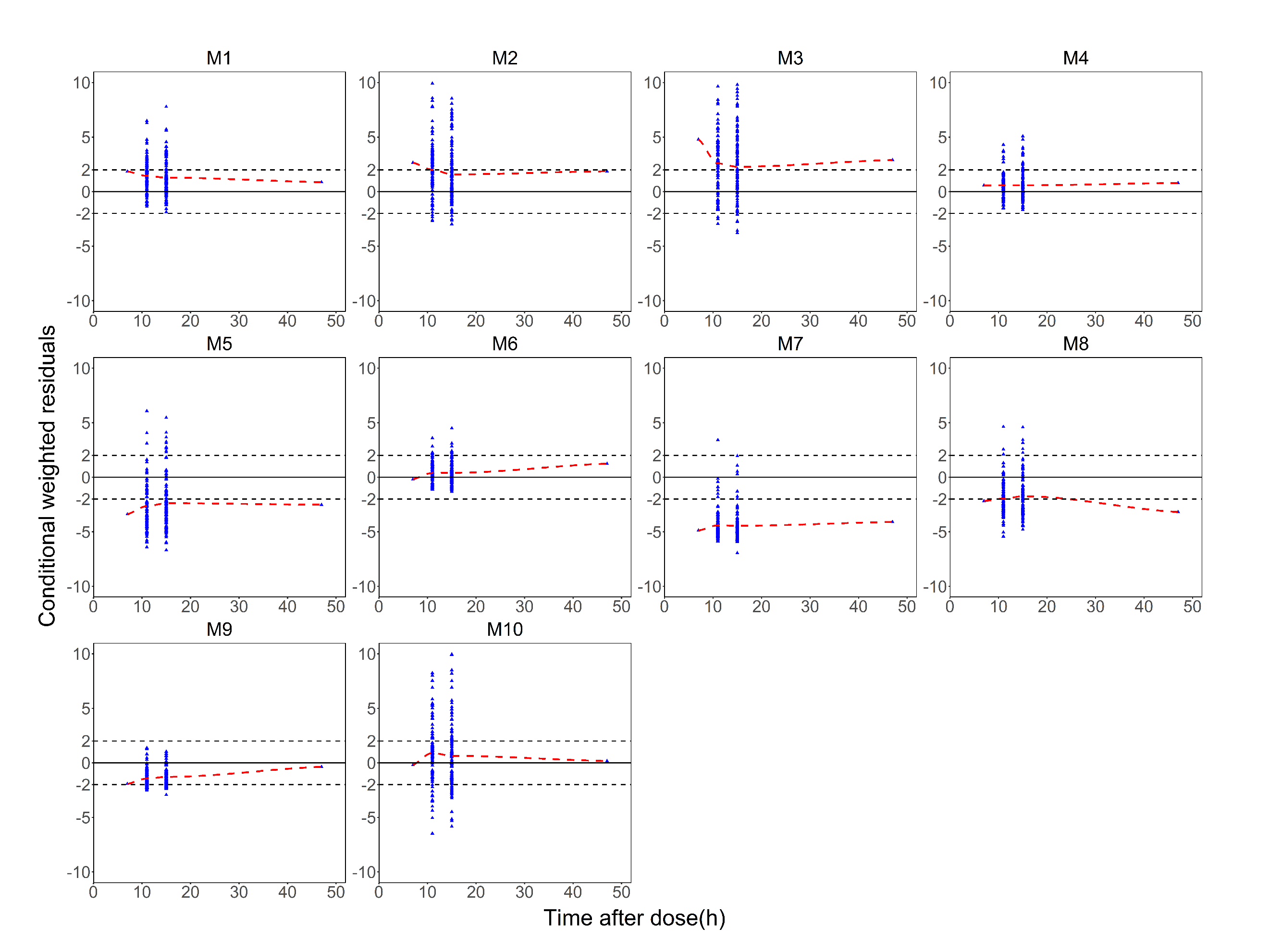


**Supplementary Figure 6.** Condition weighted residuals versus time after dose of 10 models evaluated with the whole cohort.





**Supplementary Figure 7.** Condition weighted residuals versus time after dose of 10 models evaluated with cohort 1 (cohort with PPI treatment in the evaluation data) and cohort 2 (cohort without PPI treatment in the evaluation data).


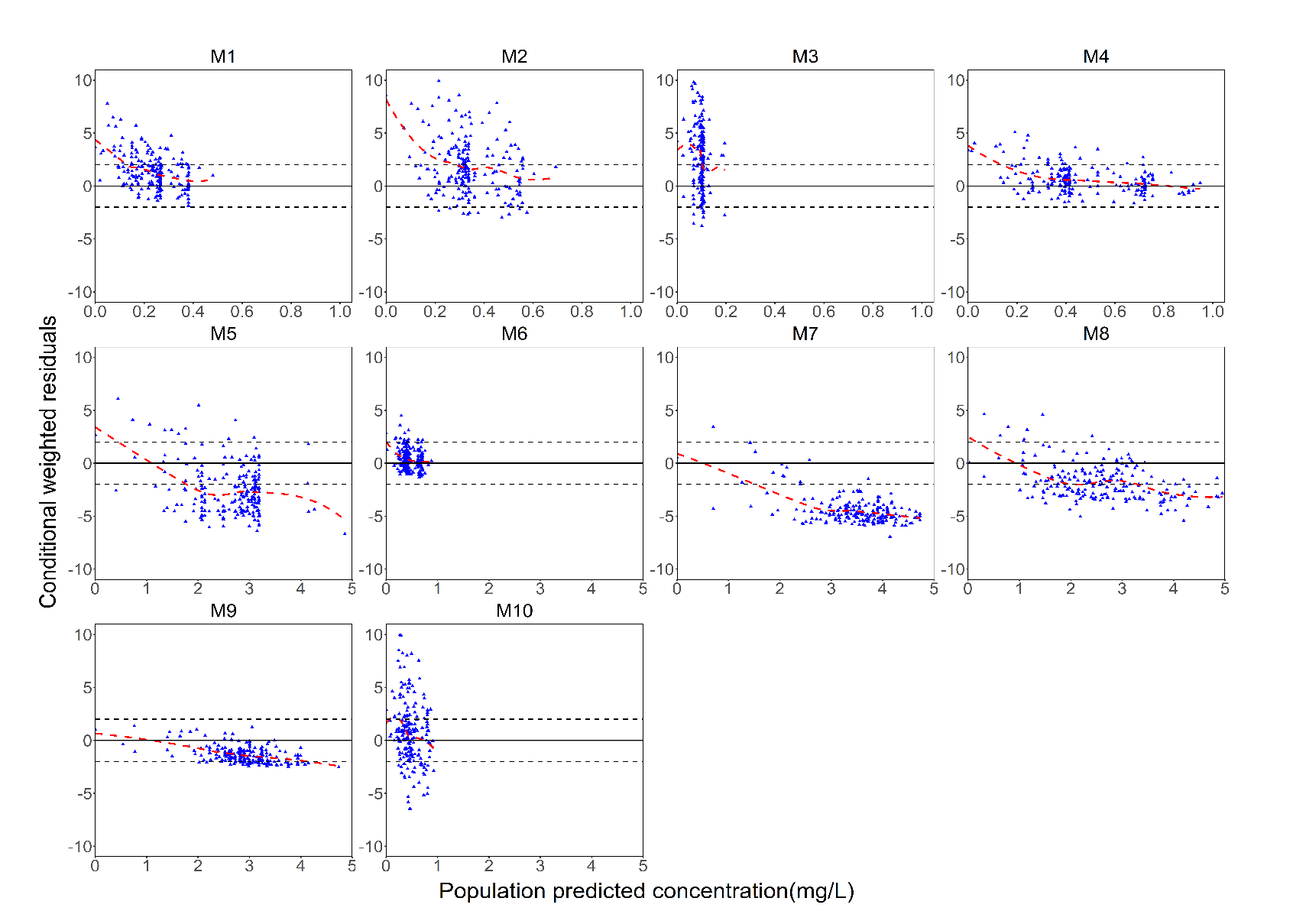


**Supplementary Figure 8.** Condition weighted residuals versus population predicted concentrations of 10 models evaluated with the whole cohort.





**Supplementary Figure 9.** Condition weighted residuals versus population predicted concentrations of 10 models evaluated with cohort 1 (cohort with PPI treatment in the evaluation data) and cohort 2 (cohort without PPI treatment in the evaluation data).


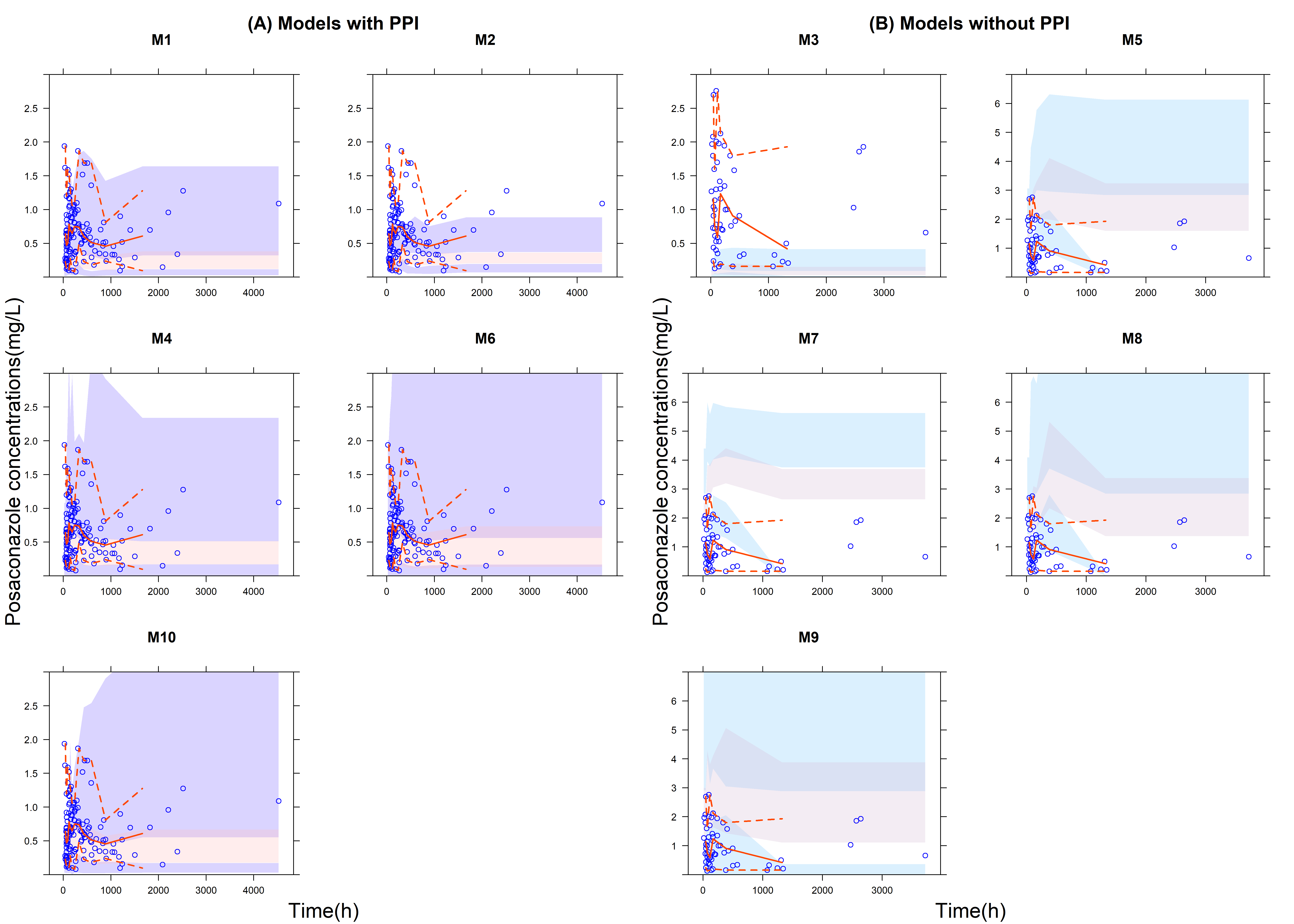


**Supplementary Figure 10.** Visual predictive check (VPC) plots for the published model of posaconazole evaluated with cohort 1 (cohort with PPI treatment in the evaluation data) and cohort 2 (cohort without PPI treatment in the evaluation data). Blue points represent the observations, and red lines represent the 5th, 50th and 95th percentiles of the observed data. The color-shaded areas represent the 95% confidence intervals around the simulated 5th, 50th, and 95th percentiles.

**Supplementary Table 1.** The results of numerical prediction error

| Models | | MPE(%) | MAPE(%) | F_20_(%) | F_30_(%) |
| --- | --- | --- | --- | --- | --- |
| M1 | whole cohort | -64.03 | 66.38 | 7.51 | 12.21 |
|  | cohort 1 | -63.67 | 65.12 | 7.19 | 11.51 |
| M2 | whole cohort | -50.45 | 55.44 | 11.27 | 17.37 |
|  | cohort 1 | -51.65 | 54.16 | 12.95 | 16.55 |
| M3 | whole cohort | -84.77 | 84.77 | 2.82 | 3.29 |
|  | cohort 2 | -89.89 | 89.89 | 0.00 | 1.72 |
| M4 | whole cohort | -35.01 | 47.58 | 15.96 | 25.35 |
|  | cohort 1 | -39.01 | 48.40 | 15.83 | 22.30 |
| M5 | whole cohort | 314.08 | 314.08 | 3.29 | 3.76 |
|  | cohort 2 | 171.33 | 171.33 | 6.90 | 8.62 |
| M6 | whole cohort | -34.32 | 49.63 | 21.60 | 27.70 |
|  | cohort 1 | -36.25 | 47.53 | 20.14 | 26.62 |
| M7 | whole cohort | 454.19 | 454.19 | 1.88 | 3.76 |
|  | cohort 2 | 250.63 | 250.63 | 8.62 | 10.34 |
| M8 | whole cohort | 295.23 | 295.23 | 1.88 | 2.35 |
|  | cohort 2 | 129.99 | 129.99 | 3.45 | 3.45 |
| M9 | whole cohort | 329.56 | 329.56 | 3.76 | 4.23 |
|  | cohort 2 | 224.02 | 224.02 | 10.34 | 12.07 |
| M10 | whole cohort | -34.05 | 48.24 | 17.84 | 29.58 |
|  | cohort 1 | -35.24 | 46.16 | 15.11 | 29.50 |

**Supplementary Table 2.** Result of Bayesian forecasting of 10 models

| Models | MIPE(%) | | | |  | MAIPE(%) | | | |  | IF_20_(%) | | | |  | IF_30_(%) | | | |
| --- | --- | --- | --- | --- | --- | --- | --- | --- | --- | --- | --- | --- | --- | --- | --- | --- | --- | --- | --- |
|  | P0 | P1 | P2 | P3 |  | P0 | P1 | P2 | P3 |  | P0 | P1 | P2 | P3 |  | P0 | P1 | P2 | P3 |
| M1 | -87.15 | -23.94 | -12.19 | -17.18 |  | 87.60 | 24.34 | 22.51 | 23.09 |  | 0.47 | 38.14 | 45.10 | 40.00 |  | 1.88 | 78.35 | 64.71 | 60.00 |
| M2 | -60.31 | -24.85 | -17.98 | -15.11 |  | 64.85 | 31.20 | 30.48 | 27.89 |  | 7.51 | 25.77 | 35.29 | 40.00 |  | 13.62 | 47.42 | 49.02 | 63.33 |
| M3 | -96.64 | -12.51 | -6.80 | -5.84 |  | 96.64 | 12.51 | 13.21 | 17.56 |  | 0.00 | 94.85 | 60.78 | 66.67 |  | 0.00 | 98.97 | 80.39 | 73.33 |
| M4 | -93.09 | -23.82 | -21.06 | -21.89 |  | 100.00 | 30.18 | 31.46 | 37.04 |  | 6.57 | 26.80 | 29.41 | 16.67 |  | 9.39 | 49.48 | 45.10 | 36.67 |
| M5 | 247.50 | 42.91 | 8.11 | 1.15 |  | 247.50 | 42.91 | 9.25 | 12.99 |  | 4.23 | 17.53 | 62.75 | 60.00 |  | 9.86 | 23.71 | 66.67 | 73.33 |
| M6 | -83.66 | -20.28 | -19.34 | -22.33 |  | 84.26 | 23.02 | 27.97 | 31.42 |  | 0.94 | 32.99 | 29.41 | 23.33 |  | 1.88 | 73.20 | 56.86 | 46.67 |
| M7 | 437.47 | 457.02 | 405.47 | 433.79 |  | 437.47 | 457.02 | 405.47 | 433.79 |  | 1.88 | 4.12 | 3.92 | 6.67 |  | 3.76 | 5.15 | 3.92 | 6.67 |
| M8 | 190.89 | 61.67 | 18.08 | 17.58 |  | 190.89 | 61.67 | 19.30 | 23.45 |  | 5.63 | 22.68 | 52.94 | 50.00 |  | 8.45 | 29.90 | 62.75 | 60.00 |
| M9 | 19.39 | 170.11 | 75.01 | 43.55 |  | 55.77 | 170.11 | 75.01 | 43.55 |  | 16.90 | 11.34 | 15.69 | 20.00 |  | 25.82 | 13.40 | 25.49 | 33.33 |
| M10 | -99.59 | -2.47 | 3.58 | 2.42 |  | 99.62 | 2.47 | 10.53 | 14.92 |  | 1.88 | 87.63 | 70.59 | 56.67 |  | 2.35 | 89.69 | 72.55 | 63.33 |
